# *Candida* colonisation in asymptomatic HIV patients attending a tertiary hospital in Benin City, Nigeria

**DOI:** 10.3402/ljm.v8i0.20322

**Published:** 2013-03-18

**Authors:** Newton O. Esebelahie, Ifeoma B. Enweani, Richard Omoregie

**Affiliations:** 1 Department of Medical Laboratory Science, Faculty of Health Sciences and Technology, College of Health Sciences, Nnamdi Azikiwe University, Nnewi Campus, Nnewi, Nigeria; 2 School of Medical Laboratory Sciences, University of Benin Teaching Hospital, Benin City, Edo State, Nigeria

**Keywords:** HAART, HAART-naive, Candida colonisation, CD4+ counts, prevalence

## Abstract

**Background:**

Candidiasis is the commonest opportunistic fungal infection in patients infected with human immunodeficiency virus (HIV). CD4+ lymphocyte counts have been found to be a marker of HIV disease progression.

**Aim:**

This study focused on determining the spectrum of *Candida* isolates in urine, stool, and oral specimens among HIV patients in a tertiary hospital.

**Methods:**

A total of 300 subjects comprising of 200 HIV patients and 100 non-HIV subjects were used for this study. Three samples (urine, stool, and oral swab) were collected from each subject. Each specimen was processed using standard microbiological techniques and emergent *Candida* isolates were identified with CHROMagar™ *Candida* and sugar fermentation tests.

**Results:**

The overall prevalence of *Candida* colonisation among HIV patients was 52.5%. HAART-naive patients had a significantly higher prevalence (OR = 3.65; 95% CI = 2.03–6.56; *p*<0.0001) than their counterpart on highly active antiretroviral therapy (HAART) (OR = 1.99; 95% CI = 1.13–3.50; *p*=0.0232). Female gender was a significant risk factor for acquiring *Candida* infection (OR = 3.40; 95% CI = 1.14–10.13; *p*=0.0289). The effect of age on prevalence of candidiasis was observed among HIV patients on HAART (*p*=0.0161). A CD4+ count <200 cells/µl was a significant risk factor for acquiring candidal infection only among HAART-naive patients (OR = 4.37; 95% CI = 1.60–11.95; *p*=0.0042). The five species of *Candida* recovered from this study were *C. albicans, C. krusei, C. parapsilosis, C. tropicalis, and C. glabrata*.

**Conclusion:**

There is a significant relationship between antiretroviral therapy, CD4+ counts, and the prevalence of candidiasis.

The human immunodeficiency virus (HIV) has emerged as a global disaster (1). HIV/AIDS continues to spread globally and remains a worldwide pandemic affecting about 40 million people (2). The pandemic is the leading cause of death in sub-Saharan Africa and the fourth leading cause of mortality worldwide, with over 95% of these deaths occurring among young adults in the developing world (3, 4). AIDS is caused by HIV and is characterised by progressive damage to the immune system, which opens the door to different opportunistic infections (5), including fungal infections. The commonly encountered fungal infections in HIV-positive patients are candidiasis, cryptococcosis, histoplasmosis, pneumocystosis, coccidioidomycosis, and penicilliosis (1, 6, 7).

The effect of fungal diseases among patients with HIV infection was recognised in the early days of the AIDS epidemic. Fungal infections were reported in many of the first patients described with a new acquired cellular immunodeficiency in 1981 (8, 9). The risk of fungal infection depends primarily on these factors: the severity of impairment of cell-mediated immunity; the risk of exposure; recent or current use of an antifungal medication; and neutropenia that relates primarily to invasive candidiasis (6).

Infections with *Candida* appear when the CD4 count is 200–500 cells/µl and may be the first indication of immunodeficiency (10). The factors contributing to the pathogenesis of *Candida* and its progression in HIV patients are poorly understood, but they may include biofilm production, an interrelationship between HIV and *Candida*, and/or dysfunction in the local immunity super-imposed on weakened cell-mediated immunity and depletion of CD4 T cells (11–13).

Highly active antiretroviral therapy (HAART) has generally been taken as the gold standard in the management of HIV patients (14). The use of HAART results in improved quality of life for HIV patients (6) as well as near normal turnover of both CD4 and CD8 T-cell populations (15–17). Therefore, it is expected that in the era of HAART, immunity of HIV patients will be improved and opportunistic infections reduced. However, oropharyngeal and oesophageal candidiasis continues to afflict HIV-infected individuals in the HAART (18–22). There are few or no reported studies on *Candida* infection among HIV patients in Benin City, Nigeria. This study was conducted against this background.

## Materials and methods

### Study area

The study was carried out in the University of Benin Teaching Hospital, Benin City, Nigeria. It is located in the South–South geopolitical zone of Nigeria. It serves as a referral hospital for about six to ten states in Nigeria. It is a centre for the Institute of Human Virology, Nigeria, and US President's Emergency Plan for AIDS Relief (PEPFAR) HIV/AIDS interventions in the zone.

### Study population

The study included 300 subjects consisting of 200 HIV patients and 100 (42 males and 58 females) apparently healthy age-matched non-HIV subjects. The patients consisted of 100 HAART-naive patients (31 males and 69 females) and 100 HIV patients on HAART for 3–6 months (22 males and 78 females). The HAART regimen included zidovudine, stavudine, and nevirapine. The HIV patients were out-patients and asymptomatic. Informed consent was obtained from all subjects prior to specimen collection. The Ethical Committee of the University of Benin Teaching Hospital approved the protocol for this study.

### Specimen collection and processing

Venous blood samples (5 ml) were collected into ethylene diamine tetraacetic acid (EDTA) containers and mixed. Stool specimens were collected in sterile wide-mouthed containers and clean-catch mid-stream urine was collected in sterile universal containers containing a few crystals of boric acid as preservative. Oral swabs were collected with sterile swab sticks.

The blood specimens were used for CD4 counts using flow cytometry (Partec, Germany) following the manufacturer's instructions.

Stool specimens were processed as previously described (23). Briefly, ∼1 g of faeces was emulsified in 10 ml of sterile water. A loopful (0.001 ml) of this was streaked on the surface of two sets of Sabouraud's dextrose agar (SDA) and brain heart infusion agar (BHIA), each containing 5 µg/ml of gentamicin. The plates were incubated at 37°C for 24–48 h. The counts were expressed in colony forming units per ml (cfu/ml). A count of ≥10^5^ cfu/ml was considered indicative of *Candida* colonisation.

A loopful (0.001 ml) of well-mixed un-centrifuged urine was streaked onto the surface of SDA and BHIA. The plates were incubated aerobically at 37°C for 24–48 h and counts were expressed in cfu/ml. A count of ≥105 cfu/ml was considered indicative of asymptomatic urinary candiduria. The urine specimens were centrifuged at 2,000 g for 5 min. The supernatant was discarded, and a drop of the deposit was examined microscopically at high magnification for pus cells. Urinary candidiasis was diagnosed if the yeast count was significant in an individual.

Oral swab was inoculated on SDA and BHIA, each containing 5 µg/ml gentamicin, and incubated at 37°C for 24–48 h. Emergent yeast colonies were stored for identification.

All *Candida* isolates were identified with CHROMagar™ *Candida* (Paris, France) and sugar fermentation test as previously described (24, 25).

## Results

HIV patients were at higher risk of acquiring *Candida* infection (HIV vs. non-HIV; OR = 2.58, 95% CI = 1.16–4.30, *p*=0.0002). However, considering the prevalence of *Candida* in relation to HIV status (HAART-naive or on HAART) and that of non-HIV subjects, only HAART-naive HIV patients had significantly higher prevalence of *Candida* colonisation (HAART-naive vs. control; OR = 3.65, 95% CI = 2.03–6.56, *p*<0.0001). In a similar vein, HAART-naive HIV patients were at higher risk of acquiring *Candida* colonisation than their counterparts on HAART (OR = 9.99, 95% CI = 1.13–93.50, *p*=0.0232; Table 1). The effect of gender on the prevalence of *Candida* colonisation among HIV and non-HIV subjects was noticed only among HIV patients on HAART, where female gender was at significant risk of acquiring *Candida* colonisation (OR = 3.40, 95% CI = 1.14–10.13, *p*=0.0289; Table 1).

**Table 1:** Prevalence of *Candida* colonisation among HIV and non-HIV subjects

	Male		Female		Total	
			
Status	No. of sampled	No. of infected (%)	No. of sampled	No. of infected (%)	No. of sampled	No. of infected (%)
Non-HIV	42	14 (33.3)	58	16 (27.6)	100	30 (30.0)
Mixed infection	42	0 (0)	58	1 (1.72)	100	1 (1.0)
HIV patients						
HAART naive^2^	31	17 (54.8)	69	44 (63.7)	100	61 (61.0)
Mixed infection	31	5 (16.3)	69	10 (14.49)	100	15 (15.0)
On HAART^1^,^3^	22	5 (22.7)	78	39 (50.0)	100	44 (44.0)
Mixed infection	22	0 (0)	78	3 (3.85)	100	3 (3.0)

HIV versus non-HIV: OR = 2.58; 95% CI = 1.160–4.30; *p*=0.0002.

1 On HAART versus control: OR = 1.83; 95% CI = 1.02–3.28; *p*=0.0569.

2 HAART naive versus control: OR = 3.65; 95% CI = 2.03–6.56; *p*<0.0001.

3 HAART naive versus on HAART: OR = 1.99, 95% CI = 1.13–3.50; *p*=0.0235.

The prevalence of *Candida* colonisation increased with age. However, this increase was only statistically significant (*p*=0.0161) among HIV patients on HAART, where the age group of 61–70 years had the highest prevalence (Table 2).

**Table 2:** Prevalence of *Candida* colonisation among HIV and non-HIV subjects in relation to age groups

Status/age group (years)	No. of sampled	No. of infected (%)	*p*
HAART-naïve			0.4206
21–30	13	9 (69.2)	
31–40	49	32 (65.3)	
42–50	33	17 (51.5)	
51–60	4	3 (75.0)	
61–70	1	0 (0.0)	
Total	100	61 (61.0)	
On HAART			0.0161
21–30	15	7 (46.7)	
31–40	44	12 (27.3)	
41–50	28	17 (60.7)	
51–60	10	05 (50.0)	
61–70	3	03 (100.0)	
Total	100	44 (44.0)	
Non-HIV			0.1592
21–30	41	10 (24.4)	
31–40	35	9 (25.7)	
41–50	16	5 (31.3)	
51–60	2	2 (100.0)	
61–70	6	4 (66.7)	
Total	100	30 (30.0)	

A CD4 count <200 cells/µl was a significant risk factor for acquiring candidiasis only among HAART-naive HIV patients (OR = 4.37, 95% CI = 1.60–11.95, *p*=0.0042; Table 3).

**Table 3:** Effect of CD4 counts on *Candida* colonisation among HIV patients

Status/CD4 count (cells/µl)	No. of sampled	No. of infected (%)	OR	95% CI	*p*
HAART-naïve					0.0055
< 200	33	27 (81.8)	4.37	1.60–11.95	
≥ 200	67	34 (50.7)	0.23	0.084–0.62	
On HAART					1.000
< 200	11	5 (45.5)	1.09	0.30–3.76	
≥ 200	89	39 (43.6)	0.94	0.27–3.30	

Five species of *Candida* were recovered. *C. albicans* was the most prevalent followed by *C. krusei* and *C. parapsilosis*, while *C. tropicalis* and *C. glabrata* were the least prevalent and were recovered only from stool specimens. *C. glabrata* was recovered only from HIV patients on HAART, while *C. albicans* was the only *Candida* species recovered from the oral cavity of HIV patients on HAART (Table 4).

**Table 4:** *Candida* isolates from HIV patients and non-HIV individuals

	Non-HIV (%)			HAART-naive (%)			On HAART (%)			
				
Isolates	Urine	Stool	Oral swab	Urine	Stool	Oral swab	Urine	Stool	Oral swab	Total
*C. albicans*	(100)	7 (87.5)	17 (94.4)	20 (86.40)	19 (65.5)	29 (87.5)	7 (87.5)	12 (68.4)	24 (100)	139 (84.2)
*C. krusei*	0	0	1 (5.6)	2 (9.1)	6 (20.6)	4 (12.5)	1 (12.5)	2 (10.5)	0	16 (9.7)
*C. parapsilosis*	0	0	0	(4.5)	3 (10.4)	0	0	1 (5.3)	0	5 (3.0)
*C. tropicalis*	0	0	0	0	1 (3.5)	0	0	1 (5.3)	0	2 (1.2)
*C. glabrata*	0	1 (12.5)	0	0	0	0	0	2 (10.5)	0	3 (1.8)

No. sampled for each clinical specimen = 100.

## Discussion

The severity of *Candida* infections increases with the number and severity of predisposing factors, such as immunodeficiency (6). Even in the HAART era, oropharyngeal and oesophageal candidiasis are still rife (22). Although reports of oral candidiasis abound, there is no report from Benin City. Against this background, this study focused on determining the spectrum of *Candida* isolates from urine, stool, and oral specimens among HIV patients in a tertiary hospital.

HIV status is a risk factor for acquiring *Candida* colonisation. This agrees with previous reports (1). However, this effect was observed only among HAART-naive HIV patients. HAART has been reported to improve immunity (14) and immunosuppression is among the factors that predispose to opportunistic *Candida* infections (1). This may explain the result in this study.

The effect of age on the prevalence of *Candida* colonisation was significant only among HIV patients on HAART, with age group 61–70 years having the highest prevalence. The reason for this significant effect in the HAART group is unclear. However, the elderly are known to have weaker immunity (25). The observed higher prevalence of *Candida* colonisation among the elderly is consistent with a recent report (13). The effect of gender on prevalence of *Candida* colonisation was observed only among female HIV patients on HAART. This may be a result of proximity of the anal region to the urinary tract in females and to hormonal imbalance.

A CD4 count <200 cells/µl was a significant risk factor for acquiring candidiasis among HAART-naive HIV patients (Table 3). This finding is supported by earlier observations of Schoots et al. (26) and Njunda et al. (22), both of whom reported a significant relationship between low CD4 counts (<200 cells/µl) and candidiasis. This was expected, as immune suppression is a hallmark of HIV infection (27).

Irrespective of HIV status and type of specimen processed, *C. albicans* was the most predominant species recovered in this study. This is in agreement with previous studies (19, 22, 28–31). Other *Candida* species recovered in this study were *C. krusei*, *C. parapsilosis*, *C. glabrata* and *C. tropicalis*. These species of *Candida* were previously reported among HIV patients (13, 19). It is important to note that only *C. krusei* and *C. glabrata* were recovered from non-HIV subjects in addition to *C. albicans*, and they were recovered from oral swab and stool specimens, respectively. This may indicate that other species of *Candida* isolated are associated with immunosuppression. It has been reported that *Candida* infections among HIV patients are refractory to antifungal agents (22), thus making this infection life-threatening among HIV patients.

## Conclusion

To sum up, candidiasis remains an important opportunistic infection among HIV patients. Measures to reduce its prevalence, such as prompt treatment with appropriate antifungals, strict compliance with HAART regimen, and reducing practices that promote yeast overgrowth, are advocated.
